# Cationic and Non-Ionic Surfactant–Assisted Morphological Engineering of CoMoO_4_ for High-Performance Asymmetric Supercapacitors

**DOI:** 10.3390/mi17010089

**Published:** 2026-01-09

**Authors:** Pritam J. Morankar, Aviraj M. Teli, Chan-Wook Jeon

**Affiliations:** 1 School of Chemical Engineering, Yeungnam University, 280 Daehak-Ro, Gyeongsan 38541, Republic of Korea; 2 Division of Electronics and Electrical Engineering, Dongguk University-Seoul, Seoul 04620, Republic of Korea

**Keywords:** cobalt molybdate, surfactant-assisted hydrothermal synthesis, CTAB/PEG morphology engineering, areal capacitance, long-term cycling stability

## Abstract

Precise morphology engineering is essential for enhancing the charge-storage capabilities of cobalt molybdate (CoMoO_4_). In this study, cobalt molybdate (CoMoO_4_, abbreviated as CoMo), cobalt molybdate–cetyltrimethylammonium bromide (CoMo-CTAB), and cobalt molybdate–cetyltrimethylammonium bromide/polyethylene glycol (CoMo-CTAB/PEG) electrodes were synthesized through a cationic–nonionic surfactant-assisted hydrothermal route. he introduction of CTAB promoted the formation of well-defined nanoflake structures, whereas the synergistic CTAB/PEG system produced a highly porous and interconnected nanosheet architecture, enabling enhanced electrolyte diffusion and redox accessibility. As a result, the CoMo-CTAB/PEG electrode delivered a high areal capacitance of 10.321 F cm^−2^ at 10 mA cm^−2^, markedly outperforming CoMo-CTAB and pristine CoMo electrodes. It also exhibited good rate capability, maintaining 63.64% of its capacitance at 50 mA cm^−2^. Long-term cycling tests revealed excellent durability, with over 83% capacitance retention after 12,000 cycles and high coulombic efficiency, indicating highly reversible Faradaic behavior. Moreover, an asymmetric pouch-type supercapacitor device (APSD) assembled using the optimized electrode demonstrated robust cycling stability. These findings underscore surfactant-directed morphology modulation as an effective and scalable strategy for developing high-performance CoMoO_4_-based supercapacitor electrodes.

## 1. Introduction

Modern energy storage research is increasingly driven by the demand for systems capable of delivering high power output while maintaining electrochemical and structural stability over prolonged operation [1,2]. Although conventional secondary batteries provide high energy density, their practical deployment is often constrained by slow charge-discharge kinetics, safety concerns under high current operation, and gradual performance degradation [3]. These shortcomings are particularly critical for applications requiring rapid energy delivery and long-term reliability. In this regard, supercapacitors have emerged as a promising alternative owing to their fast response, long cycle life, and superior safety characteristics [4,5]. Nevertheless, the relatively low energy density of supercapacitors remains a significant limitation, highlighting the urgent need for advanced electrode materials that can simultaneously achieve high power capability and enhanced energy storage [6]. The electrochemical performance of supercapacitors is fundamentally governed by the nature of the electrode materials and their charge storage mechanisms [7]. Carbon-based materials such as activated carbon, graphene, and carbon nanotubes are widely used due to their high electrical conductivity and excellent cycling stability; however, their energy storage mechanism is primarily based on non-Faradaic electrostatic ion adsorption, which inherently limits their capacitance [8]. Conductive polymers provide higher capacitance through Faradaic redox reactions, but their practical application is hindered by mechanical degradation and volume changes during repeated cycling [9]. In contrast, transition metal oxides offer rich redox chemistry and high theoretical capacitance, making them attractive for pseudocapacitive energy storage. Despite these advantages, their practical performance is often limited by poor electrical conductivity and sluggish ion diffusion kinetics [10].

To overcome these challenges, binary transition metal oxides have attracted considerable attention, as the presence of two electrochemically active metal cations enables synergistic redox behavior and improved charge transport [11]. Among them, metal molybdates are particularly appealing due to the flexible valence states of molybdenum and its ability to stabilize the crystal structure during reversible redox processes [12]. Enhanced electrochemical performance has been reported for several molybdates, including NiMoO_4_, MnMoO_4_, FeMoO_4_, and Bi_2_MoO_6_, which benefit from cooperative redox activity and favorable electronic coupling within the molybdate lattice. Within this family, CoMoO_4_ has emerged as a promising pseudocapacitive material due to the coexistence of multivalent cobalt and molybdenum ions [13]. The availability of Co^2+/^Co^3+^/Co^4+^ and Mo^4+^/Mo^6+^ redox couples enable efficient and reversible Faradaic reactions, while strong electronic interactions between cobalt and molybdenum centers facilitate rapid charge transfer [12,14,15].

Recent studies have demonstrated that the electrochemical performance of CoMoO_4_ is strongly dependent on its morphology and structural architecture. Pitcheri et al. reported ultra-small, rice-like one-dimensional CoMoO_4_ nanorods synthesized via a one-pot solvothermal method, achieving a high specific capacitance of 1608.8 F g^−1^ with excellent cycling stability and good rate capability [13]. Chen et al. developed petal-like CoMoO_4_ clusters grown on carbon cloth as a self-standing, binder-free electrode, delivering a specific capacitance of 664 F g^−1^ and high energy density in assembled supercapacitor devices [16]. Sivakumar et al. demonstrated vertically aligned porous CoMoO_4_ nanoflakes, where optimized heat treatment at 400 °C resulted in a high specific capacity and superior energy and power densities in a hybrid supercapacitor [17]. Barmi et al. demonstrated that the non-ionic surfactant Pluronic F127 effectively tailors the surface of CoMoO_4_, transforming rod-like structures into nanospheres with improved pore accessibility and conductivity, resulting in a specific capacitance of 79 F g^−1^ and ~80% retention after 2000 cycles [18]. In contrast, Hou et al. showed that the cationic surfactant CTMAB enables the formation of ultrafine 1D CoMoO_4_·0.9H_2_O nanorods with weak crystallinity and high surface area, delivering a much higher capacitance of 377 F g^−1^ with ~93% stability after 1000 cycles [19]. Despite these promising advances, the electrochemical performance of CoMoO_4_ frequently remains below its theoretical potential, limited by insufficient rate capability, partial utilization of active sites, and progressive capacity fading during long-term cycling [20]. These limitations are often associated with dense or poorly accessible morphologies, agglomeration of active particles, and structural stress generated during repeated redox reactions. Therefore, precise control over nucleation, crystal growth, and surface architecture during synthesis is crucial for unlocking the intrinsic battery-type Faradaic properties of CoMoO_4_ [21].

Among various synthesis strategies, surfactant-assisted hydrothermal routes provide an effective platform for tailoring the morphology and electrochemical accessibility of transition metal oxides. In particular, cationic and non-ionic surfactants influence nucleation and growth through fundamentally different mechanisms. Cationic surfactants such as CTAB regulate crystal growth via electrostatic interactions with precursor ions, promoting controlled nucleation and anisotropic architectures. In contrast, non-ionic surfactants such as PEG act as soft templates and steric stabilizers that suppress agglomeration, enhance porosity, and facilitate electrolyte penetration [22]. In this work, surfactant chemistry is deliberately employed as a decisive synthesis parameter to engineer CoMoO_4_ nanostructures. Pristine CoMo, CoMo-CTAB, and CoMo-CTAB/PEG are systematically synthesized via a hydrothermal route. The distinct roles of cationic and non-ionic surfactants in regulating morphology, surface accessibility, and ion transport kinetics are critically examined. By establishing clear correlations between surfactant-induced structural evolution and electrochemical behavior, this study elucidates an effective strategy for maximizing active-site utilization and redox efficiency in CoMoO_4_ electrodes. Furthermore, the optimized CoMoO_4_ electrode is integrated into an asymmetric supercapacitor configuration to demonstrate its practical applicability for high-performance energy storage systems.

## 2. Experimental Section

### 2.1. Materials

Cobalt nitrate hexahydrate (Co(NO_3_)_2_·6H_2_O), ammonium heptamolybdate tetrahydrate ((NH_4_)_6_Mo_7_O_24_·4H_2_O), cetyltrimethylammonium bromide (CTAB), polyethylene glycol (PEG), polyvinylidene fluoride (PVDF), and N-methyl-2-pyrrolidone (NMP) were purchased from Sigma-Aldrich and used as received without further purification. Nickel foam (1 × 2 cm^2^) was employed as the current collector for electrode preparation. Deionized (DI) water was used for all synthesis and washing procedures.

### 2.2. Synthesis of Pristine and Surfactant-Modified CoMoO_4_ Nanostructures

**Figure 1:**
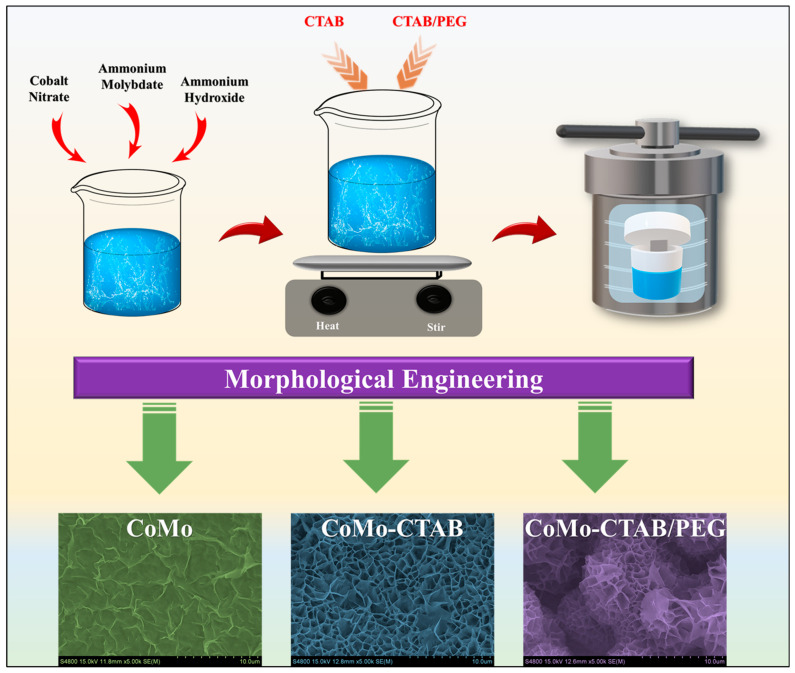
Schematic illustration of the surfactant-assisted hydrothermal process used for synthesizing CoMoO_4_ electrodes.

CoMoO_4_ powders were synthesized via a hydrothermal route with controlled surfactant incorporation to tailor morphology, porosity, and electrochemical accessibility. For all samples, 2 mmol of Co(NO_3_)_2_·6H_2_O and 1.33 mmol of (NH_4_)6Mo_7_O_24_·4H_2_O were dissolved in 80 mL of DI water under magnetic stirring until a homogeneous solution was obtained. The pH was adjusted to approximately 7–8 using dilute NH_4_OH to promote uniform hydrolysis and controlled nucleation. For the pristine CoMoO_4_ sample, no surfactant was added. For the CTAB-assisted sample, 0.01 wt% of CTAB was introduced to the precursor solution and stirred for 30 min to direct anisotropic crystal growth. In the combined cationic and non-ionic surfactant system, 0.005 wt% of CTAB and 0.005 wt% of PEG were added together, where CTAB promoted directional growth while PEG acted as a steric stabilizer to prevent aggregation and enhance porosity. The resulting solutions were transferred to 100 mL Teflon-lined stainless-steel autoclaves and heated at 180 °C for 12 h. After natural cooling to room temperature, the powders were collected, washed thoroughly with DI water and ethanol, and dried at 60 °C for 12 h. Finally, the powders were annealed at 400 °C for 2 h in air to obtain crystalline CoMoO_4_ with optimized morphology and structural stability. Figure 1 illustrates the schematic representation of the synthesis process, showing the stepwise formation pathway and how surfactant-assisted modifications guide the structural evolution from pristine densely aggregated particles to hierarchical nanoarchitectures in the CoMo, CoMo-CTAB, and CoMo-CTAB/PEG samples.

### 2.3. Electrode Fabrication

Electrodes were fabricated using the CoMo, CoMo-CTAB, and CoMo-CTAB/PEG powders. Each powder was mixed with conductive carbon black and PVDF binder in a weight ratio of 80:10:10, and NMP was added to form a uniform slurry. The slurry (2.0 mg cm^−2^) was then coated onto pre-cleaned nickel foam (1 × 2 cm^2^) and dried at 80 °C for 12 h under vacuum. The coated electrodes were lightly pressed to improve adhesion and directly used for electrochemical measurements in a three-electrode configuration.

## 3. Material Characterization and Electrochemical Measurements

The synthesized CoMo, CoMo-CTAB, and CoMo-CTAB/PEG samples were characterized using a comprehensive set of analytical techniques. X-ray diffraction (XRD, PANalytical diffractometer with Cu Kα radiation, λ = 1.5406 Å) was performed to determine the crystal structure and phase purity of the materials. Field-emission scanning electron microscopy (FE-SEM, S-4800, HITACHI, Chiyoda, Tokyo, Japan) was employed to examine the surface morphology and nanostructural features at various magnifications. Samples were sputter-coated with platinum prior to imaging to minimize charging artifacts. Energy-dispersive X-ray spectroscopy (EDS) integrated with the FE-SEM was used to analyze the elemental composition and spatial distribution of cobalt, molybdenum, and oxygen throughout the samples. For electrochemical characterization, a Biologic WBCS3000 potentiostat (BioLogic Science Instruments, Seyssinet-Pariset, France) was used in a three-electrode configuration with the CoMo-based electrode on nickel foam as the working electrode, platinum foil as the counter electrode, and Ag/AgCl as the reference electrode in 2 M KOH aqueous electrolyte. Cyclic voltammetry (CV) was performed to assess the capacitive behavior and electrochemical reversibility of the materials. Galvanostatic charge–discharge (GCD) measurements were conducted to determine the specific capacitance and rate performance at various current densities. Electrochemical impedance spectroscopy (EIS) was used to evaluate the charge-transfer resistance and electrochemical kinetics of the electrode materials. Long-term cycling tests were performed to evaluate the stability and durability of the electrodes. Finally, the optimized CoMo-CTAB/PEG electrode was assembled into an asymmetric supercapacitor device using activated carbon as the complementary electrode, and the device performance was evaluated in terms of specific capacitance, energy density, power density, and cycling stability.

## 4. Results and Discussion

### 4.1. X-Ray Diffraction Elucidation

**Figure 2:**
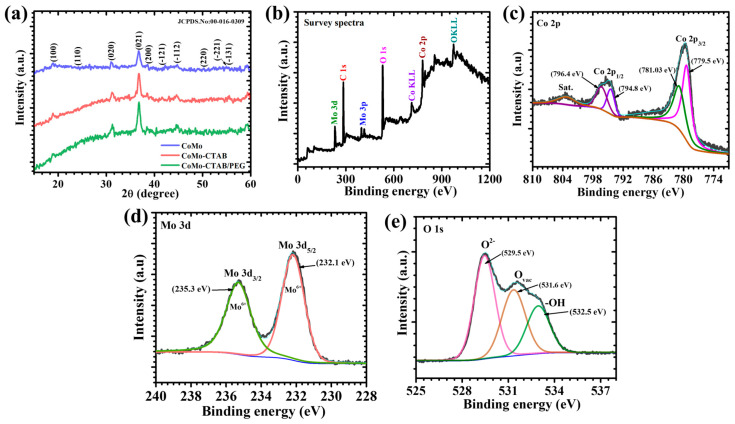
(**a**) XRD patterns of CoMo, CoMo-CTAB, and CoMo-CTAB/PEG electrodes. XPS analysis of the optimized CoMo-CTAB/PEG electrode: (**b**) survey spectrum, (**c**) Co 2p, (**d**) Mo 3d, and (**e**) O 1s high-resolution spectra.

The XRD patterns of the CoMo, CoMo-CTAB, and CoMo-CTAB/PEG samples are presented in Figure 2a. The diffraction peaks match well with the monoclinic CoMoO_4_ phase (JCPDS No: 00-016-0309), confirming that the target compound has been successfully synthesized in all three samples. Importantly, the same set of characteristic CoMoO_4_ diffraction peaks indexed to the (100), (110), (020), (021), (200), (-121), (-112), (220), and (-131) planes is observed in CoMo, CoMo-CTAB, and CoMo-CTAB/PEG samples, indicating identical crystallographic phase formation. The pristine CoMo sample exhibits sharp, well-defined peaks that indicate good long-range crystalline order. The CoMo-CTAB and CoMo-CTAB/PEG samples show similar peak positions, confirming that the crystal structure remains unchanged despite the surfactant modifications [23]. However, the intensity and sharpness of the peaks differ slightly among the samples, with the surfactant-modified samples showing somewhat broadened peaks compared to the pristine CoMo sample. These differences in peak characteristics reflect the influence of the synthesis conditions on the crystal development. The surfactants used in the synthesis likely affect the nucleation and growth process, leading to variations in the structural features of the final products. The consistent indexing of all peaks to the CoMoO_4_ phase across all three samples demonstrates that the surfactant additions do not cause unwanted phase transformations or contamination. Instead, they serve their intended purpose of controlling morphology while preserving the desired crystalline structure. This result confirms that our synthesis approach successfully produces phase-pure CoMoO_4_ materials with well-defined crystal structures suitable for battery-type Faradaic electrode applications [24].

### 4.2. X-Ray Photoelectron Spectroscopy Elucidation

To gain deeper insight into the surface elemental composition and oxidation states of the optimized CoMo-CTAB/PEG sample, X-ray photoelectron spectroscopy (XPS) analysis was carried out. The XPS survey spectrum (Figure 2b) confirms the presence of Co, Mo, and O elements without any detectable impurity signals, indicating the successful formation of phase-pure CoMoO_4_. The high-resolution spectra of Co 2p, Mo 3d, and O 1s are presented in Figure 2c–e, respectively. The Co 2p spectrum (Figure 2b) displays two principal peaks located at 779.5 eV (Co 2p_3/2_) and 794.8 eV (Co 2p_1/2_), along with distinct satellite features at 781.0 and 796.4 eV The deconvolution clearly reveals contributions from both Co^2+^ and Co^3+^ species, indicating the coexistence of mixed-valence cobalt states. The presence of mixed cobalt oxidation states (Co^2+/^Co^3+^) provides multiple accessible redox transitions, which play a vital role in enhancing battery-type Faradaic charge-storage kinetics. The Mo 3d spectrum (Figure 2c) displays a sharp and symmetric doublet associated exclusively with Mo^6+^, consisting of well-resolved peaks at 231.2 eV (Mo 3d_5/2_) and 235.3 eV (Mo 3d_3/2_). The absence of any lower-binding-energy shoulders confirms that molybdenum remains in a fully oxidized hexavalent state without the presence of Mo^4+^ or Mo^5+^ species. This stable Mo^6+^ environment is consistent with the formation of pure CoMoO_4_ spinel-type coordination in the final composite [25]. The O 1s spectrum (Figure 2d) can be deconvoluted into three components: a major peak at ~529.5 eV assigned to lattice oxygen (O^2−^), a peak at ~531.6 eV associated with oxygen vacancies or defect oxygen species, and a higher binding energy peak at ~532.5 eV corresponding to surface hydroxyl groups (–OH). The prominent contribution of defect oxygen indicates the presence of abundant oxygen vacancies created during synthesis, which play a crucial role in improving surface redox activity and accelerating electrolyte ion diffusion. Meanwhile, the –OH related peak reflects the surface hydroxylation, which enhances electrolyte wetting and further facilitates rapid charge transport. The XPS results confirm that the CoMo-CTAB/PEG sample contains a favorable combination of mixed-valence Co and Mo states along with a high density of oxygen vacancies. These features synergistically contribute to improved electrical conductivity, enhanced redox reversibility, and superior interfacial ion transport which directly supporting the excellent electrochemical performance observed for this optimized electrode [26].

### 4.3. Morphological and Elemental Compositional Characteristics

**Figure 3:**
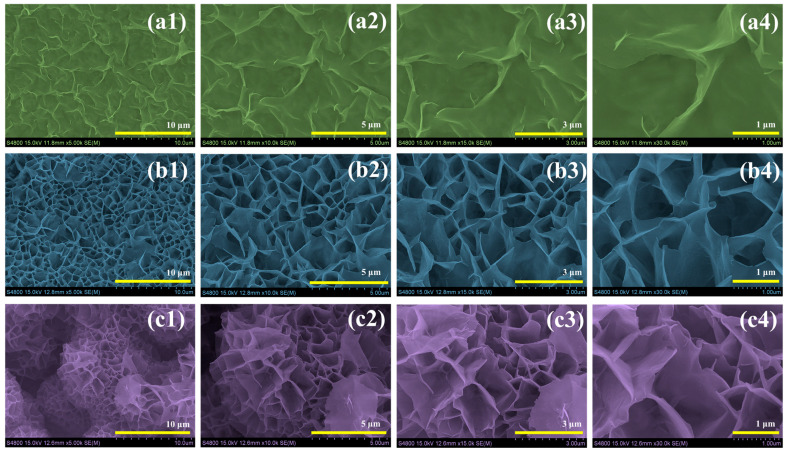
FESEM images of (**a1**–**a4**) CoMo, (**b1**–**b4**) CoMo-CTAB, and (**c1**–**c4**) CoMo-CTAB/PEG samples recorded at different magnifications.

The morphological features of the CoMo, CoMo-CTAB, and CoMo-CTAB/PEG samples were characterized using FESEM. Figure 3 shows the FESEM images recorded at different magnifications, which reveal significant structural differences among the three samples and highlight the impact of surfactant chemistry on morphology control. The pristine CoMo sample Figure 3(a1–a4) shows a relatively flat, sheet-like surface with poor definition and minimal porosity. The material exhibits dense particle aggregation with limited nanostructural features. This compact structure limits the available surface area and hinders effective electrolyte penetration into the material, thereby restricting access to active redox sites. As a result, the electrochemical performance of this sample is compromised. When CTAB is introduced as a structure-directing agent, the CoMo-CTAB sample Figure 3(b1–b4) displays a markedly transformed morphology. The cationic surfactant promotes the formation of interconnected nanosheets that are more open and porous compared to the pristine sample. The nanosheets are thin, regularly distributed, and feature enhanced surface roughness. This arrangement creates multiple pathways for electrolyte ion transport and substantially increases the effective surface area available for electrochemical reactions. The resulting nanostructure clearly shows improved accessibility for ion diffusion and interfacial charge transfer. The CoMo-CTAB/PEG sample Figure 3(c1–c4) exhibits the most optimized nanostructure among the three samples. The synergistic effect of both CTAB and PEG results in a hierarchical architecture characterized by thinner, well-separated nanosheets with uniform spacing. The porosity appears more refined and better organized at multiple scales. The addition of PEG, a non-ionic polymer, functions as an effective stabilizer that suppresses agglomeration and enhances pore development, leading to an overall more favorable structure for electrolyte accessibility. The clear morphological evolution observed from the pristine to the surfactant-modified samples demonstrates the importance of surfactant engineering in material design. The progression begins with dense aggregates in CoMo, followed by interconnected porous sheets in CoMo-CTAB, and culminates in a well-organized hierarchical nanostructure in CoMo-CTAB/PEG. The superior morphology achieved in CoMo-CTAB/PEG, with its optimized porosity and enhanced surface accessibility, directly supports the enhanced electrochemical performance observed in the subsequent supercapacitor measurements [21,27].

**Figure 4:**
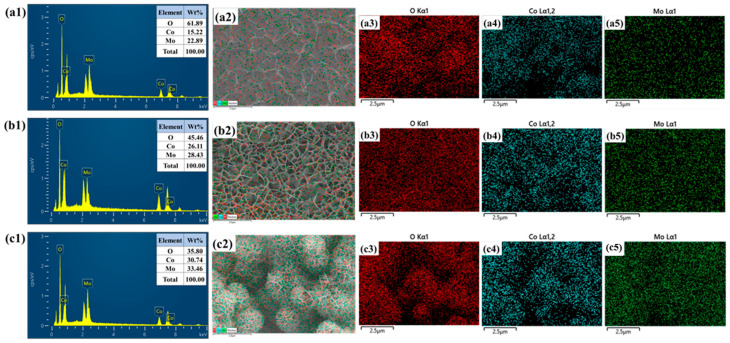
(**a1**–**c1**) EDS spectra, (**a2**–**c2**) FESEM–EDS elemental overlays, and (**a3**–**c5**) Elemental mapping images of the CoMoO_4_ samples.

The elemental composition and distribution of the CoMo, CoMo-CTAB, and CoMo-CTAB/PEG samples were examined through EDS and elemental mapping, and the results are shown in Figure 4. The EDS spectra Figure 4(a1,b1,c1) verify the presence of Co, Mo, and O in all three materials, confirming the successful formation of the cobalt molybdate phase. A noticeable change in the elemental ratios is observed across the series, with the CoMo-CTAB/PEG sample showing relatively higher Co and Mo contents compared to the pristine CoMo. This trend agrees well with the improved structural definition and greater exposure of active sites achieved through the combined CTAB and PEG assistance. The FESEM overlays Figure 4(a2,b2,c2) further support the morphological evolution seen in the FESEM images, where the pristine CoMo displays a compact, densely stacked sheet-like surface. With CTAB, the structure becomes more open and develops into interconnected porous nanosheets, while the CoMo-CTAB/PEG sample shows a more refined hierarchical structure made up of thin nanosheets together with small nanosheet-to-nanosphere features. The elemental mapping images Figure 4(a3–a5,b3–b5,c3–c5) show that Co, Mo, and O are uniformly distributed throughout each sample, indicating that the surfactants do not cause elemental segregation. The mapping for the CoMo-CTAB/PEG sample, in particular, highlights a very even and dense distribution of all elements, which reflects its highly accessible and porous architecture. Such a well-developed structure provides more exposed redox-active sites and is consistent with the improved electrochemical performance observed for this material.

### 4.4. Electrochemical Analysis

**Figure 5:**
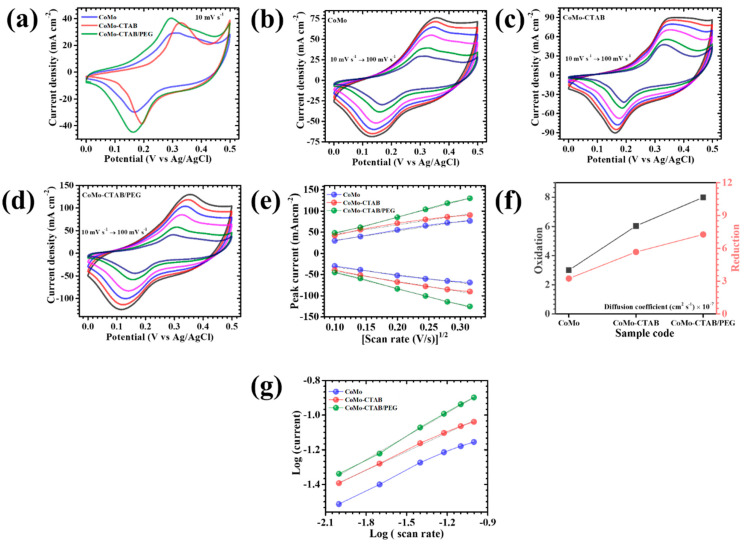
(**a**) Cyclic voltammetry (CV) curves of all CoMoO_4_ electrodes at a scan rate of 10 mV s^−1^ in the potential window of 0–0.5 V; CV curves at different scan rates for (**b**) CoMo, (**c**) CoMo-CTAB, and (**d**) CoMo-CTAB/PEG; (**e**) peak current vs. (scan rate)^1/2^ plot; (**f**) diffusion coefficient calculation; (**g**) log(i) vs. log(ν) plot.

The electrochemical performance of the CoMo-based electrodes synthesized using different surface-modifying agents CTAB and PEG, was systematically evaluated through CV, GCD, and EIS in a standard three-electrode configuration with 2 M KOH electrolyte. Figure 5a displays the CV curves of pristine CoMo, CoMo-CTAB, and the hybrid CoMo-CTAB/PEG electrodes recorded at 10 mV s^−1^ within the potential window of 0–0.5 V. The introduction of CTAB and the CTAB/PEG mixture brings about significant changes in the redox features and overall capacitive response of the electrodes, demonstrating the critical influence of surfactant-directed synthesis on physicochemical properties. Such improvements arise from the ability of these surfactants, particularly the CTAB/PEG hybrid, to modulate nucleation behavior and growth rate during material formation, thereby altering the surface topology, crystallinity, and abundance of electrochemically active sites. To further examine the rate capability and reaction reversibility, CV measurements were conducted across a range of scan rates from 10 to 100 mV s^−1^, as shown in Figure 5b–d. All samples exhibit broad and distinguishable redox couples with oxidation peaks near ~0.32 V and reduction peaks around 0.1–0.15 V, which align well with the typical Faradaic transitions of Co^2+^/Co^3+^ and Co^3+^/Co^4+^ [28]. The non-rectangular shape of the CV profiles, coupled with the appearance of pronounced redox signals, clearly confirms that charge storage is dominated by battery-type Faradaic behavior associated with reversible surface or near-surface redox pathways, rather than by purely electrostatic double-layer processes. These redox transitions arise from reversible OH^−^ insertion and extraction within the CoMoO_4_ matrix, described by the following reaction (1) [29,30]:

(1) $$CoMoO_{4}+{OH}^{-}⇌\;CoOOH+{MoO}_{3}+e^{-}$$

The CV curves recorded at different scan rates show only minor deformation as the scan rate increases, indicating strong redox reversibility and stable ion-interaction kinetics. Slight anodic and cathodic peak shifts are observed, which are typical and mainly attributed to increased polarization at higher sweep rates. Nonetheless, the consistent of peak symmetry and overall curve shape implies efficient OH^−^ transport and consistent Faradaic activity. The superior behavior of the CoMo-CTAB/PEG sample can be linked to the finely tuned morphology generated by the synergistic action of CTAB and PEG during synthesis. Surfactant-driven control over particle assembly minimizes agglomeration, enhances surface roughness, and creates an interconnected porous network. This optimized nanoflower-like architecture provides abundant ion-accessible channels, maximizes electroactive surface sites, and establishes continuous electron pathways. In contrast, the surfactant-free CoMo electrode forms a more compact, low-porosity structure in which ion mobility is restricted, limiting the number of accessible redox centers and reducing charge-transfer rates. Although the CoMo-CTAB sample shows moderate improvement, its partially dense microstructure still impedes electrolyte diffusion and slows the kinetics of redox reactions. Consequently, the dual-surfactant-engineered CoMo-CTAB/PEG electrode delivers the most favorable electrochemical attributes in terms of structural openness, electrical connectivity, and utilization of active sites, reinforcing its role as the optimized composition among all investigated samples [31].

To further elucidate the charge-transfer mechanism and ion diffusion behavior in the CoMo-based electrodes, CV was conducted over a wide range of scan rates. As depicted in Figure 5e, the anodic and cathodic peak currents (*i_p_*) exhibit a strong linear correlation with the square root of the scan rate (*v*^1/2^) for all electrode compositions. This linearity confirms that the redox processes are primarily diffusion-limited, a typical signature of well-defined Faradaic reactions where ion transport through the electrode-electrolyte interface governs the overall kinetics. To quantitatively assess ion diffusion capability, the apparent diffusion coefficients (D) were estimated using the Randles–Sevcik Equation (2) [32]:

(2) $$D^{1/2}=\frac{i_{p}}{2.69\times 10^{5}\times n^{3/2}\times A\times C\times v^{1/2}}$$

In this expression, *n* represents the number of electrons involved in the redox event, *A* denotes the effective electrochemical surface area, *C* is the concentration of the redox-active species in the electrolyte, and *v* corresponds to the applied scan rate. The calculated diffusion coefficients for all electrodes at 10 mV s^−1^ are summarized in Table 1, with additional comparative visualization provided in Figure 5f. Among the three studied materials, the CoMo-CTAB/PEG electrode exhibits the highest D value, signifying superior ionic diffusion and accelerated charge-transfer activity. This enhancement arises from the hierarchical and highly open nanoflower-like framework produced through the cooperative action of CTAB and PEG, which generates more accessible pathways for electrolyte penetration and minimizes solid-state diffusion resistance. In contrast, the bare CoMo and single-surfactant CoMo-CTAB electrodes display comparatively lower diffusion coefficients. The poorest diffusion characteristics observed for pristine CoMo can be attributed to its compact, less-developed morphology formed in the absence of any surfactant, which restricts ion movement and reduces exposure of active sites essential for efficient Faradaic reactions [33].

Underlying charge-storage behavior of the CoMo-based electrode kinetics were evaluated using the power-law relationship ($i={av}^{b}$), which correlates the peak current (*i*) with the applied scan rate (*v*). The exponent b derived from this relation stands for critical descriptor of the governing charge-storage mechanism. A b value near 0.5 reflects a diffusion-limited Faradaic process, while values approaching 1 signify a surface-controlled capacitive response [34]. By plotting *log(i) against log(v)*, as shown in Figure 5g, the b values were extracted and summarized in Table 1. All electrodes exhibited b values in the range of 0.35–0.50, clearly establishing that their energy-storage behavior arises predominantly from diffusion-mediated redox reactions, although minor capacitive contributions are also present.

To more precisely unravel the diffusion-driven and surface-controlled fractions of the current response, the CV data were further interpreted using the kinetic separation model expressed in Equation (3) [35]:

(3) $$i\left(V\right)=k_{1}v{+k_{2}v}^{1/2}$$

**Figure 6:**
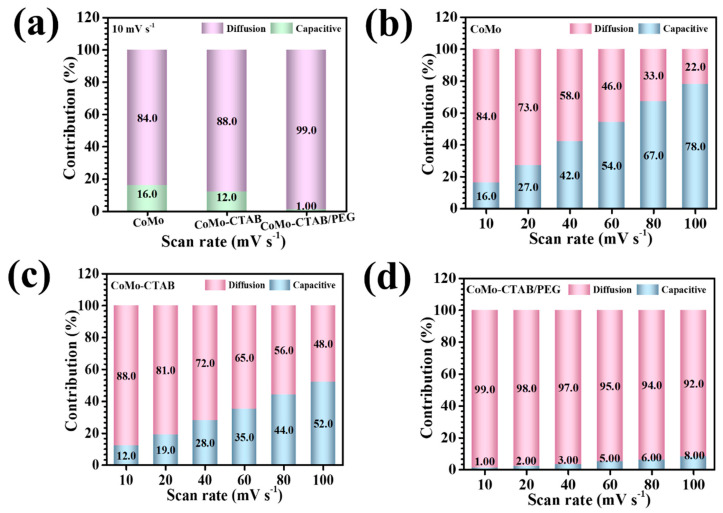
Capacitive and diffusion-controlled charge-storage contributions: (**a**) comparison of all CoMoO_4_ electrodes at 10 mV s^−1^, (**b**) CoMo at various scan rates, (**c**) CoMo-CTAB, and (**d**) CoMo-CTAB/PEG.

Here, *k*_1_*v* denotes the capacitive component predominantly originating from rapid surface-level interactions, while *k*_2_*v*^1/2^ corresponds to the diffusion-governed Faradaic contribution. These constants were extracted through linear fitting of *i(V)/v*^1/2^ against *v*^1/2^, enabling accurate quantification of each mechanism. Using this approach, the total accumulated charge can be resolved as indicated in Equation (4) [35]:

(4) $$Q_{t}=Q_{s}+Q_{d}$$

where *Q_s_* represents the capacitive share, and *Q_d_* accounts for diffusion-controlled redox contributions. At a scan rate of 10 mV s^−1^, the capacitive-to-diffusion contributions for the CoMo, CoMo-CTAB, and CoMo-CTAB/PEG electrodes were determined as 16/84%, 12/88%, and 1/99%, respectively (Figure 6a). These results clearly reveal that the integration of CTAB/PEG significantly amplifies the diffusion-dominated charge-storage process within the CoMoO_4_ lattice. Remarkably, the CoMo-CTAB/PEG electrode exhibited an exceptionally high diffusion contribution of ~99% at a lower scan rate of 1 mV s^−1^, indicating superior ion-transport capability and highly favorable electrochemical reaction kinetics. Such performance is attributed to its well-developed hierarchical nanoarchitecture, consisting of interconnected nanosheets forming an open, porous flower-like network. This structural design enhances electrolyte permeation, increases the density of accessible redox-active sites, and provides efficient pathways for ion migration. The influence of scan rate on the relative contributions of capacitive and diffusion-controlled processes was further analyzed across the range of 10–100 mV s^−1^. As shown in Figure 6b–d for CoMo, CoMo-CTAB, and CoMo-CTAB/PEG, a consistent rise in the capacitive fraction was observed with increasing scan rate for all electrodes. This shift occurs because higher scan rates limit the penetration of electrolyte ions into deeper active regions, thus favoring rapid surface-driven charge accumulation. The growing dominance of capacitive behavior at elevated sweep rates highlights the importance of an optimized electrode microstructure that can support fast ion interactions while maintaining strong redox activity. The hybrid-surfactant-engineered CoMo-CTAB/PEG electrode achieves this balance most effectively, which is reflected in its superior rate performance and overall electrochemical efficiency [36,37].

**Figure 7:**
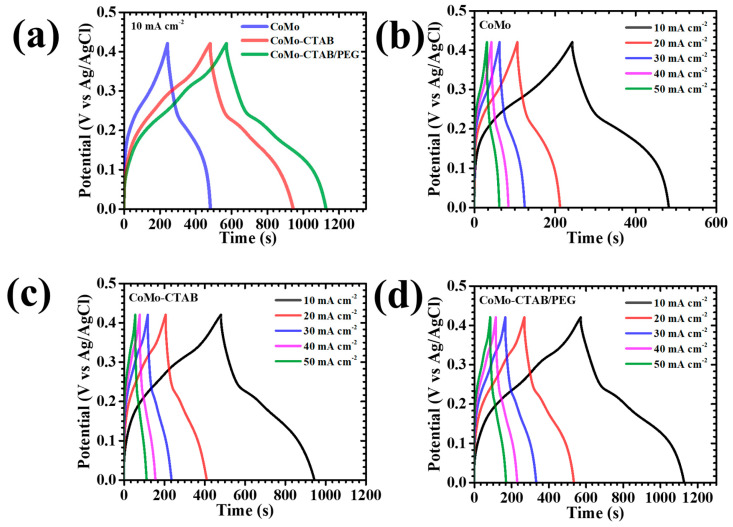
(**a**) GCD curves of all CoMoO_4_ electrodes at 10 mA cm^−2^; GCD profiles at various current densities for (**b**) CoMo, (**c**) CoMo-CTAB, and (**d**) CoMo-CTAB/PEG.

**Figure 8:**
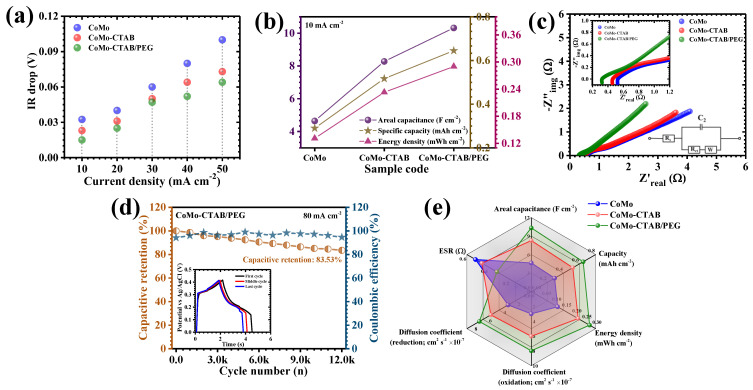
(**a**) IR-drop analysis and comparison of areal capacitance, specific capacity, and energy density for CoMoO_4_ electrodes; (**b**) Nyquist (EIS) plots of the electrodes; (**c**) long-term cycling stability of the CoMo-CTAB/PEG electrode over 12,000 GCD cycles (Inset: comparative GCD curves from the initial, middle and final cycles); (**d**) Long-term cycling stability of the optimized CoMo-CTAB/PEG electrode at 80 mA cm^−2^ over 12,000 cycles, with representative GCD profiles from the initial, middle, and final cycles; (**e**) Multidimensional performance comparison of the CoMo, CoMo-CTAB, and CoMo-CTAB/PEG electrodes.

Figure 7a presents the GCD characteristics of the CoMo, CoMo-CTAB, and CoMo-CTAB/PEG electrodes obtained at a constant current density of 10 mA cm^−2^ within the potential range of 0–0.42 V. All samples reveal distinctly nonlinear charge-discharge curves featuring prominent voltage plateaus, which are hallmarks of diffusion-controlled Faradaic reactions typical of battery-type Faradaic materials [38]. Among the investigated electrodes, the CoMo-CTAB/PEG sample exhibits a more prominent and an exceptionally smooth discharge trace, suggesting highly reversible OH^−^ intercalation and rapid redox transformations at the active interface [38]. Moreover, the significantly prolonged discharge time of CoMo-CTAB/PEG relative to CoMo and CoMo-CTAB confirms its noticeably enhanced charge-storage ability, directly associated to the hierarchical nanostructure engineered through the synergistic effect of CTAB and PEG during synthesis. Current-dependent GCD profiles recorded between 10 and 50 mA cm^−2^ (Figure 7b–d) further support these observations. Across the entire current range, all electrodes retain their characteristic voltage plateaus consistent with the Faradaic redox couples identified in the CV study, reaffirming the dominance of Faradaic charge-storage mechanisms. The charge-discharge curves of each electrode display near-symmetric shapes, indicative of high Coulombic efficiency, minimal polarization losses, and stable, reversible redox kinetics. Notably, the CoMo-CTAB/PEG electrode demonstrates the best symmetry and an exceptionally small IR drop at the start of discharge, signifying superior electronic conductivity and accelerated ion-transport dynamics. The nearly linear portion of its GCD curves further reflects its robust structural framework capable of withstanding continuous redox cycling without degradation. Additional insight into resistance behavior is provided by the IR-drop analysis in Figure 8a. A consistent decrease in IR drop is observed for all electrodes as the applied current density is reduced, confirming diminished resistive contributions at slower discharge rates. Throughout the entire current range, the CoMo-CTAB/PEG electrode registers the lowest IR-drop values, underscoring its significantly reduced internal resistance and highly efficient interfacial charge-transfer capability.

A comprehensive quantitative understanding of the electrodes was performed through the areal capacitance (C_A_), energy density (ED), and power density (PD), and they were calculated using the following equations, which accurately describe the nonlinear discharge characteristics typical of pseudocapacitive systems (5)–(7) [39,40]:

(5) $$C_{A}=\frac{I\times 2\times \int V\left(t\right)dt}{A\times {\left(∆V\right)}^{2}}$$

(6) $$ED=\frac{1}{2\times 3600}\;C_{A}\times {\left(∆V\right)}^{2}$$

(7) $$PD=\frac{ED\times 3600}{T_{d}}$$

In these expressions, *I* is the discharge current, *∫V(t)dt* represents the integrated discharge area, *A* is the geometric electrode area, *ΔV* is the operational voltage window, and *T_d_* is the discharge period. These formulations enable a more accurate evaluation of performance by incorporating the intrinsic nonlinear discharge behavior of Faradaic electrodes. At 10 mA cm^−2^, the areal capacitances of the CoMo, CoMo-CTAB, and CoMo-CTAB/PEG electrodes were determined to be 4.642, 8.277, and 10.321 F cm^−2^, respectively (Table 2, Figure 8b). The exceptionally high capacitance obtained for the CoMo-CTAB/PEG material highlights the effectiveness of using both CTAB and PEG as co-structuring agents. This enhancement can be attributed to the formation of a highly porous, flower-like network that increases the density of active sites, improves electrolyte penetration, and shortens ion-diffusion pathways due to its interconnected channels. The influence of increasing current density on capacitance and energy density, summarized in Table 2, reveals a consistent decline for all electrodes. This reduction is characteristic of restricted OH^−^ diffusion at higher current loads, where only surface-accessible sites contribute significantly to charge storage [28]. Despite the expected decrease, the CoMo-CTAB/PEG electrode maintains 63.64% of its initial capacitance at 50 mA cm^−2^, demonstrating remarkable rate capability and mechanical robustness under demanding electrochemical conditions. Overall, the comprehensive GCD analysis demonstrates that the CoMo-CTAB/PEG electrode possesses a highly optimized electrochemical architecture distinguished by enhanced conductivity, reduced polarization, abundant electroactive surface area, and rapid ion/electron transport. The deliberate integration of CTAB and PEG during synthesis enables a synergistic balance between structural uniformity, porosity, and mechanical strength. Consequently, the hybrid CoMo-CTAB/PEG material delivers far superior charge-storage performance and high-rate stability compared with pristine CoMo and its single-surfactant-modified counterpart.

EIS was conducted to gain a deeper understanding of the intrinsic charge-transport behavior and interfacial electrochemical characteristics of the CoMo-based electrodes. The corresponding Nyquist plots, obtained across a broad frequency range spanning 10 kHz to 0.1 Hz in 2 M KOH electrolyte (Figure 8c), offer detailed information regarding ionic resistance in the electrolyte, interfacial charge-transfer processes, and ion-diffusion dynamics within the electrode matrix. Electrochemical impedance spectra of the CoMo, CoMo-CTAB, and CoMo-CTAB/PEG electrodes were quantitatively interpreted by fitting the experimental Nyquist plots using a classical equivalent circuit, denoted as (R1 + C2/R2 + W3), where R1 represents the series resistance, C2 corresponds to the interfacial double-layer capacitance, R2 denotes the charge-transfer resistance, and W accounts for the Warburg diffusion element. The fitting procedure was carried out using EC-Lab software v11.32, and the extracted parameters are summarized in Table 1. For the pristine CoMo electrode, the fitted values of R1, C2, R2, and W were 0.53 Ω, 0.3453 F, 2.336 Ω, and 1.0341 Ω·s-0.5, respectively. The CoMo-CTAB electrode exhibited reduced resistive components, with R1 and R2 decreasing to 0.46 Ω and 1.1272 Ω, accompanied by an increase in C2 to 0.918 F and a lower Warburg coefficient of 0.7103 Ω·s-0.5. Notably, the CoMo-CTAB/PEG electrode displayed the most favorable impedance characteristics, featuring the lowest series resistance (R1 = 0.32 Ω), the highest interfacial capacitance (C2 = 1.0104 F), the smallest charge-transfer resistance (R2 = 0.691 Ω), and the lowest Warburg coefficient (0.663 Ω·s-0.5). The progressive reduction in R1 and R2 across the electrode series indicates increasingly efficient electron transport and accelerated Faradaic charge-transfer kinetics, while the substantial enhancement in C2 reflects improved interfacial charge-storage capability and electrolyte accessibility. Collectively, the fitted EIS results confirm that hybrid CTAB/PEG-assisted synthesis effectively optimizes both charge-transfer and ion-transport processes, providing quantitative support for the superior electrochemical kinetics [41,42].

To evaluate the long-term electrochemical resilience of the optimized electrode, extensive cycling stability measurements were performed on the CoMo-CTAB/PEG sample under demanding conditions involving 12,000 successive charge–discharge cycles at a high current density of 80 mA cm^−2^ (Figure 8d), with comparative GCD profiles from the initial, middle and final cycles provided in the inset. Impressively, the electrode preserved nearly 83.53% of its initial capacitance after prolonged cycling, corresponding to a minimal degradation of approximately 16.47%. This outstanding retention clearly demonstrates the remarkable durability and strong reversibility of the redox processes occurring within the hybrid-surfactant-modified CoMoO_4_ framework. The exceptional cycling performance can be directly linked to the well-defined hierarchical nano-flower morphology produced through the synergistic effects of CTAB and PEG during synthesis. This intricately organized structure provides ample free volume to buffer mechanical stresses associated with repeated ion insertion and extraction, facilitates unobstructed electrolyte penetration, and enables rapid ionic transport throughout the porous matrix. Additionally, the electrode maintained a high coulombic efficiency exceeding 94.6% throughout the 12,000-cycle test, signaling highly reversible Faradaic reactions and negligible influence from parasitic side reactions or resistive losses. Such high reversibility indicates that the structural integrity and accessibility of electroactive sites remain largely intact even under strenuous operational conditions. The slight reduction in capacitance observed after extensive cycling may be attributed to gradual ion entrapment, wherein OH^−^ ions become immobilized within narrow porous channels or accumulate at internal interfaces over time. This ion accumulation can partially hinder the active sites required for reversible redox activity, thereby contributing to a modest decline in overall capacitance [43]. Despite this minor limitation, the electrode still demonstrates excellent endurance and stable performance over repeated cycling. Overall, these findings highlight the critical importance of deliberate morphological and interfacial engineering in designing advanced battery-type Faradaic materials. The CoMo-CTAB/PEG electrode exemplifies how strategic regulation of surface chemistry and structural topology can profoundly enhance long-term cycling reliability, sustain high-rate performance, and improve overall charge-storage efficiency.

The radar diagram presented in Figure 8e offers a comprehensive multidimensional comparison of the key electrochemical performance indicators- areal capacitance, specific capacity, energy density, ion-diffusion coefficient, and ESR for the CoMo, CoMo-CTAB, and CoMo-CTAB/PEG electrodes. This integrated visualization clearly highlights the superior and well-balanced electrochemical characteristics of the CoMo-CTAB/PEG electrode. Its expanded radial coverage across all evaluated parameters reflects the successful integration of high charge-storage capability, enhanced ionic diffusion kinetics, and significantly reduced internal resistance within a single, optimally engineered electrode. Such a uniformly strong performance profile reinforces the effectiveness of the dual-surfactant strategy in achieving a structurally and electrochemically optimized CoMoO_4_ architecture.

A comparative FESEM analysis of the optimized CoMo-CTAB/PEG electrode was conducted after 12,000 GCD cycles, reveals a hierarchical nanosheet-based flower-like architecture composed of densely interconnected, ultrathin flakes (Figure S1a,b), providing abundant electroactive surface area and open ion-diffusion pathways. Importantly, post-cycling FESEM images acquired after long-term stability testing show that this nanosheet framework is largely preserved, with no evident structural collapse or severe agglomeration, indicating strong mechanical integrity during repeated charge-discharge operation. The retained porous morphology after cycling corroborates the high capacitance retention and stable coulombic efficiency, confirming that the CoMo-CTAB/PEG electrode maintains structural and electrochemical stability under prolonged electrochemical stress.

## 5. Electrochemical Performance of Asymmetric Supercapacitor Device

**Figure 9:**
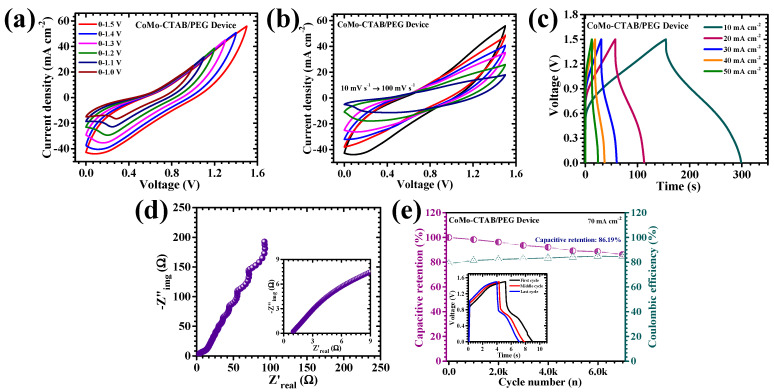
(**a**) CV curves of the CoMo-CTAB/PEG//AC hybrid device recorded within potential windows ranging from 0–1.0 V to 0–1.5 V; (**b**) CV curves of the device at scan rates from 10–100 mV s^−1^ (0–1.5 V); (**c**) GCD curves at different current densities; (**d**) EIS analysis; (**e**) long-term cycling stability over 7000 GCD cycles (Inset: GCD curves from the initial, middle and final cycles).

The practical feasibility and device-level performance of the optimized CoMo-CTAB/PEG electrode was further validated by assembling an asymmetric pouch-type supercapacitor device (APSD) using CoMo-CTAB/PEG as the Faradaic positive electrode and activated carbon (AC) as the electric double-layer negative electrode. This electrode pairing strategically exploits the battery-type pseudocapacitance of CoMoO_4_ and the high surface-area-driven charge accumulation capability of AC, thereby enabling a high-energy, high-rate asymmetric configuration. Both electrodes were uniformly coated onto nickel foam substrates (mass loading 2 mg cm^−2^) to ensure excellent electrical contact, superior mechanical integrity, and reliable operational stability. A 2 M KOH-soaked separator (Whatman filter paper separator (thickness ~100 µm)) was inserted between the electrodes, followed by sealing to preserve moisture levels and prevent environmental interference. The pouch-type device was assembled by sequentially stacking the positive electrode, electrolyte-soaked separator, and negative electrode, followed by airtight sealing to prevent electrolyte evaporation and environmental contamination. The CoMo-CTAB/PEG electrode exhibited a reliable working range of 0–0.5 V, while the AC electrode performed optimally within −1.0–0 V. These complementary operating margins enabled the CoMo-CTAB/PEG//AC device to function safely within an expanded voltage window of 1.5 V. Subsequent CV analyses carried out across different voltage windows from 1.0 to 1.5 V (Figure 9a) displayed clear battery-type Faradaic signatures with distinct redox peaks, confirming the stable electrochemical behavior across wide voltage range in the full device. As the scan rate increased from 10 to 100 mV s^−1^ (Figure 9b), the CV curves demonstrated progressively increasing currents without significant distortion, indicating rapid ionic diffusion, preserved structural integrity, and excellent electrochemical reversibility at high operational rates. The GCD curves of the assembled APSD obtained at variable current densities from 10 to 50 mA cm^−2^ (Figure 9c) further corroborate its battery-type Faradaic nature. The nonlinear discharge profiles and consistent plateaus validate the participation of Faradaic reactions within the CoMo-CTAB/PEG electrode during device operation. At a current density of 10 mA cm^−2^, the device delivered an areal capacitance of 0.476 F cm^−2^, accompanied by corresponding energy and power densities of 0.149 mWh cm^−2^ and 1.90 mW cm^−2^, respectively (Table 3). These performance levels highlight the ability of the asymmetric device to maintain a desirable balance between high energy output and fast charge-discharge capability, key attributes for real-world supercapacitor applications.

The internal resistance characteristics of the APSD were examined through Nyquist analysis (Figure 9d). The impedance spectrum features a small semicircle at high frequencies, followed by an inclined Warburg line at low frequencies, suggesting low charge-transfer resistance and unobstructed ion diffusion. The extremely low ESR of 0.98 Ω confirms highly efficient electron transport pathways and minimal ohmic losses, which can be attributed to the orderly hierarchical nanoarchitecture of the CoMo-CTAB/PEG electrode and to the strong interfacial compatibility with the AC counter-electrode. Long-term cycling durability of the APSD was rigorously evaluated through 7000 consecutive charge-discharge cycles conducted at a high current density of 70 mA cm^−2^ (Figure 9e), along with the initial, middle, and final GCD curves. The device retained 86.19% of its original capacitance after extended cycling, demonstrating robust operational stability and excellent structural resilience. The outstanding cycling endurance originates from the mechanically adaptive nanoflower-like CoMo-CTAB/PEG morphology, which accommodates volumetric changes during repeated redox cycling, thereby minimizing structural degradation and maintaining uninterrupted ion transport. Overall, the CoMo-CTAB/PEG-based APSD exhibits an exceptional combination of (i) high areal capacitance, (ii) a wide operational voltage window, (iii) low internal resistance, and (iv) excellent long-term cycling stability. These attributes collectively underscore the immense potential of the CoMo-CTAB/PEG electrode for integration into advanced, high-performance supercapacitor technologies. The remarkable improvements achieved through hybrid CTAB/PEG surfactant engineering highlight the significance of controlled nanoscale morphology and interfacial design in developing next-generation energy-storage devices optimized for practical applications.

A comparative assessment of the electrochemical performance of CoMoO_4_-based electrodes reported in the literature and the present study is summarized in Table 4. Chen et al. [17] reported petal-like CoMoO_4_ clusters grown on carbon cloth, delivering a high gravimetric capacitance of 664 F g^−1^ and an energy density of 27 Wh kg^−1^; however, the cycling stability was limited to 84% retention after 1000 cycles. Sivakumar et al. [18] demonstrated vertically aligned CoMoO_4_ nanoflakes with improved ion diffusion pathways, achieving 102 F g^−1^ in a hybrid device with 91.86% retention after 10,000 cycles. Barmi et al. [18] introduced F127-assisted CoMoO_4_ nanospheres, which improved pore accessibility and conductivity, resulting in 79 F g^−1^ and 80% capacitance retention after 2000 cycles. Tao et al. [21] developed a CoMoO_4_@CuCo_2_O_4_ core–shell architecture exhibiting an exceptionally high gravimetric capacitance of 2639 F g^−1^, although the performance was evaluated mainly in gravimetric terms. Similarly, Zhou et al. [21] reported NiO flakes@ CoMoO_4_ core–shell electrodes with 1097 F g^−1^ and excellent cycling stability (97.5% after 2000 cycles). In contrast to these reports, the CoMo-CTAB/PEG electrode developed in this work exhibits a markedly higher areal capacitance of 10.321 F cm^−2^, along with a superior areal energy density of 0.290 mWh cm^−2^ and stable power delivery of 1.88 mW cm^−2^. Moreover, it demonstrates long-term cycling durability with 83% capacitance retention after 12,000 cycles, outperforming most reported CoMoO_4_-based electrodes in terms of areal performance and operational stability. This enhanced performance is attributed to the synergistic role of CTAB and PEG in constructing a porous, interconnected nanosheet architecture, which facilitates efficient ion diffusion, abundant electroactive sites, and robust charge-transfer kinetics. These results clearly establish surfactant-assisted morphology engineering as an effective strategy for achieving high-performance CoMoO_4_-based supercapacitor electrodes.

## 6. Conclusions

This work establishes a clear structure–performance correlation for CoMoO_4_ based electrodes through cationic–nonionic surfactant engineering. The pristine CoMo displayed limited ion accessibility, whereas CoMo-CTAB developed into more open nanoflakes with improved electroactivity. The combined CTAB/PEG system produced a highly porous nanosheet network in CoMo-CTAB/PEG, enabling rapid ion diffusion, efficient utilization of redox sites, and superior electrochemical performance. Consequently, CoMo-CTAB/PEG exhibited the highest areal capacitance, excellent rate retention, and remarkable cycling durability, maintaining 83.53% capacitance over 12,000 cycles with coulombic efficiency exceeding 94.6%. The corresponding APSD further demonstrated outstanding long-term stability, sustaining 86.19% capacitance after 7000 cycles, confirming the mechanical and structural robustness of the optimized architecture. Overall, this study provides a convincing demonstration that surfactant-assisted morphological tuning is an effective, scalable, and reliable strategy for advancing high-capacitance and long-life energy-storage electrodes.

## Figures and Tables

**Table 1:** Estimated Diffusion coefficient, b-values, and series resistance values of CoMo, CoMo-CTAB, CoMo-CTAB/PEG samples.

Sample Code	Diffusion Coefficient (cm^2^/s) × 10^−7^		b-Value	R1 (Ω)	R2 (Ω)
	Oxidation	Reduction			
**CoMo**	3	3.24	0.36	0.53	2.336
**CoMo-** **CTAB**	6.035	5.664	0.39	0.46	1.1272
**CoMo-** **CTAB/PEG**	7.991	7.264	0.49	0.32	0.691

**Table 2:** Evaluation of calculated areal capacitance, specific capacity, energy density, and power density values of CoMo, CoMo-CTAB, CoMo-CTAB/PEG electrodes.

Sample Code	I (mA cm^−2^)	Areal capacitance C_A_ (F cm^−2^)	Capacity (mAh cm^−2^)	Energy Density ED (mWh cm^−2^)	Power Density PD (mW cm^−2^)
**CoMo**	10	4.642	0.290	0.131	1.97
	20	3.951	0.247	0.111	3.81
	30	3.259	0.204	0.092	5.32
	40	2.884	0.180	0.081	6.89
	50	2.568	0.160	0.072	8.50
**CoMo-** **CTAB**	10	8.277	0.517	0.233	1.81
	20	6.677	0.417	0.188	3.31
	30	5.570	0.348	0.157	4.85
	40	4.741	0.296	0.133	6.19
	50	4.099	0.256	0.115	7.55
**CoMo-** **CTAB/PEG**	10	10.321	0.645	0.290	1.88
	20	9.402	0.588	0.264	3.57
	30	8.356	0.522	0.235	5.13
	40	7.427	0.464	0.209	6.57
	50	6.568	0.410	0.185	7.92

**Table 3:** Calculated energy storage parameters of CoMo-CTAB/PEG//AC asymmetric pouch-type supercapacitor device.

Sample Code	I (mA)	CA (F cm^−2^)	C (mAh cm^−2^)	ED (mWh cm^−2^)	PD (mW cm^−2^)
**CoMo-CTAB/PEG device**	10	0.476	0.099	0.149	1.90
	20	0.331	0.069	0.103	3.39
	30	0.241	0.050	0.075	4.53
	40	0.178	0.037	0.056	5.56
	50	0.127	0.026	0.040	6.04

**Table 4:** Comparative assessment of energy storage performance of reported data with current study.

Material	Experimental Method	Specific Capacitance (F cm^−2^)	Energy Density (mWh/cm^2^)	Power Density (mW/cm^2^)	Cyclic Stability	References
CoMoO_4_	Hydrothermal Synthesis	1.328	0.054	1.20	84.0% retention after 1000 cycles	[16]
CoMoO_4_	Hydrothermal Synthesis	0.204	0.063	38.58	91.86% retention after 10,000 cycles	[17]
F127-modified CoMoO_4_	Hydrothermal Synthesis	0.158	0.076	-	80% retention after 2000 cycles	[18]
CoMoO_4_@CuCo_2_O_4_	Hydrothermal Synthesis		0.102	1.60	91% retention after 2000 cycles	[20]
NiO flakes@CoMoO_4_	Hydrothermal Synthesis	2.194	0.052	1.79	97.5% retention after 2000 cycles	[21]
**CoMo-CTAB/PEG**	**Hydrothermal Synthesis**	**10.321**	**0.290**	**1.88 mW**	**83% retention after 12,000 cycles**	**This work**

## Data Availability

The data presented in this study are available on request from the corresponding author.

## Supplementary Materials

The following supporting information can be downloaded at: https://www.mdpi.com/article/10.3390/mi17010089/s1, Figure S1: (a,b) FESEM images of CoMo-CTAB/PEG electrode after long-term cycling stability.
