# Supplementary material for: Cationic and Non-Ionic Surfactant–Assisted Morphological Engineering of CoMoO4 for High-Performance Asymmetric Supercapacitors
Source: Micromachines (Basel). 2026 Jan 9;17(1):89. doi: 10.3390/mi17010089 (PMC12843681; doi:10.3390/mi17010089)
Supplement: Supplementary file 1 [file micromachines-17-00089-s001.zip › micromachines-4069417-supplementary.pdf]

micromachines

MDPI

*supplementary materials*

# Cationic and Non-Ionic Surfactant–Assisted Morphological Engineering of $CoMoO_4$ for High-Performance Asymmetric Supercapacitors

**Pritam J. Morankar, Aviraj M. Teli and Chan-Wook Jeon**

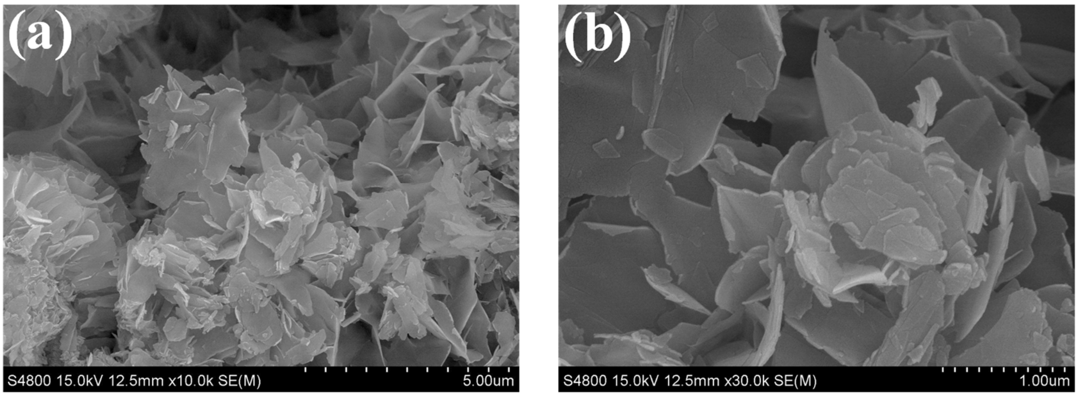


**Figure S1.** (**a**,**b**) FESEM images of CoMo-CTAB/PEG electrode after long-term cycling stability.

 https://doi.org/10.3390/mi17010067
